# Digital dentistry among clinical-year dental students: awareness, attitudes, and curriculum expectations

**DOI:** 10.1186/s12909-026-09901-5

**Published:** 2026-07-15

**Authors:** İbrahim Tevfik Gülşen, Elif Gamze Yıldız Gökşen, Etem Tayfun Gökşen, Enes Mustafa Aşar, Ayşe Meşe

**Affiliations:** 1 Department of Oral and Maxillofacial Radiology, Faculty of Dentistry, Gaziantep University, Üniversite Blv., Gaziantep, 27310 Türkiye; 2 Department of Prosthodontics, Faculty of Dentistry, Dicle University, Diyarbakır, Türkiye; 3 Department of Pediatric Dentistry, Faculty of Dentistry, Selçuk University, Konya, Türkiye

**Keywords:** Digital dentistry, Dental education, Undergraduate dental students, Curriculum development, Digital technology, Student attitudes

## Abstract

**Background:**

Digital technologies — including computer-aided design and manufacturing systems, three-dimensional printing, digital radiography, intraoral scanners, and artificial intelligence — are increasingly central to clinical dentistry, yet their integration into undergraduate curricula remains uneven. Clinical-year students, approaching graduation and patient-facing practice, are a critical group in whom to assess this transition, but evidence on how they perceive these technologies and relate them to curriculum expectations is limited. This study evaluated the awareness, attitudes, and curriculum expectations of clinical-year dental students regarding digital dentistry.

**Methods:**

A descriptive cross-sectional survey was conducted among fourth- and fifth-year students at a single dental faculty using a census-based approach. An 11-item online questionnaire assessed familiarity with five digital technologies, attitudes, and future professional perspectives, with an attention-check item and electronic informed consent. The questionnaire underwent multi-step validation, including expert-panel content validity, exploratory factor analysis, and internal-consistency reliability. Group comparisons by sex and year of study were examined with chi-square tests and Cramér’s V, applying the Benjamini–Hochberg procedure to control the false discovery rate.

**Results:**

Of 187 included students (85.0% response rate), the questionnaire showed strong content validity (scale-level content validity index = 0.94) and acceptable reliability (Cronbach’s alpha = 0.83 for familiarity and 0.92 for the positive-attitude scale). Familiarity was highest for digital radiography and lowest for artificial intelligence. Attitudes were strongly positive: most students reported high interest in digital dentistry and a clear desire for greater curricular coverage, and 74.3% considered digital dentistry teaching at their faculty insufficient. Apparent differences by sex and year of study did not remain statistically significant after correction for multiple comparisons.

**Conclusions:**

Clinical-year dental students held favourable attitudes toward digital dentistry and expressed strong demand for expanded training, yet their self-reported familiarity remained modest and was lowest for artificial intelligence. This gap between motivation and perceived provision highlights a concrete target for curriculum development, suggesting that undergraduate dental education should strengthen structured, hands-on exposure to digital technologies — particularly artificial intelligence — to better prepare graduates for contemporary clinical practice.

**Supplementary Information:**

The online version contains supplementary material available at https://doi.org/10.1186/s12909-026-09901-5.

## Background

Digital technologies are increasingly adopted across many fields to improve efficiency and accessibility. In healthcare, they support crucial activities such as monitoring, diagnosing, and treating patients, and have progressively benefited several disciplines including dentistry, where digital tools have provided faster, more precise, and more patient-centred solutions than conventional approaches [1, 2].

Several key technologies have contributed to the digital transformation of dentistry, including digital intraoral scanners, computer-aided design and computer-aided manufacturing (CAD/CAM) systems, three-dimensional (3D) printing technologies, digital radiographic systems, and artificial intelligence-supported diagnostic tools. By reshaping diagnostic, restorative, prosthetic, and educational workflows, these technologies have become increasingly relevant to undergraduate dental education [3, 4]. Given that contemporary students use digital tools in daily life, dental education is expected to evolve in parallel with this shift. Although many dental schools have begun to offer digital dentistry courses, the integration of digital technologies into undergraduate curricula remains an ongoing process rather than a completed transformation [5].

Among the core components of digital dentistry, CAD/CAM systems have played a particularly important role by enabling the digital design and fabrication of dental restorations. Since their introduction in the late twentieth century, these systems have evolved remarkably and are now considered an integral part of modern dental treatment [6]. In addition, 3D printing has grown rapidly in dentistry, and its combination with CAD/CAM workflows and digital imaging—particularly cone-beam computed tomography (CBCT) and virtual planning—has enabled the production of patient-specific surgical guides and dental models [7]. Artificial intelligence (AI) has also become an increasingly important component of digital dentistry, with applications ranging from radiographic image analysis and diagnostic support to treatment planning and the design of dental restorations. In dental education, AI-based tools, including chatbots, adaptive learning platforms, and generative systems, may support personalised learning, clinical reasoning, and timely feedback [8].

In Türkiye, the relevance of digital dentistry should also be considered in relation to the structure of the dental workforce and the growing role of private dental practice. According to recent national data [9], most actively practising dentists work in the private sector, and the number of private oral and dental healthcare institutions is substantial. In parallel, Ministry of Health data indicate that more than one thousand oral and dental healthcare facilities have been authorised to provide international health tourism services [10]. Health tourism in dentistry is commonly associated with patient expectations for timely, aesthetic, standardised, and cost-effective treatments, all of which may increase the demand for digitally supported workflows [11–13]. In this increasingly competitive and internationally oriented clinical environment, such workflows may contribute to more efficient treatment planning, communication, documentation, and service delivery. Therefore, undergraduate dental education should prepare students not only to recognise these technologies but also to understand their role in contemporary clinical practice.

Despite the growing importance of digital dentistry in modern dental care, undergraduate dental students may differ in their level of awareness, previous exposure, and confidence regarding these technologies. This variation may be particularly important among clinical-year students, who are approaching graduation and are more likely to encounter digital systems in patient care. However, evidence remains limited regarding how these students perceive multiple core components of digital dentistry together and how their perceptions relate to expectations for curriculum development. Therefore, this study aimed to evaluate the awareness, attitudes, and curriculum expectations of clinical-year dental students regarding digital dentistry. The study focused on students’ familiarity with CAD/CAM systems, 3D printing technologies, digital radiographic systems, intraoral scanners, and artificial intelligence applications, as well as their views on the role of these technologies in undergraduate dental education and future clinical practice.

We hypothesised that clinical-year students would show generally favourable attitudes towards digital dentistry, but that their self-reported familiarity with its core technologies would be uneven, reflecting the variable extent to which each technology is embedded in routine clinical training.

## Materials and methods

### Study design and ethical considerations

This descriptive cross-sectional survey study was conducted at Dicle University Faculty of Dentistry, Türkiye. Fourth- and fifth-year dental students were selected as the target population because they were in the clinical phase of undergraduate dental education and were therefore more likely to encounter digital technologies in diagnostic, restorative, prosthetic, and treatment-planning workflows. Although both cohorts follow the same curriculum, the two clinical years differ in the amount of accumulated hands-on clinical experience and in their proximity to graduation and independent practice. Comparing them was therefore intended not to test a predefined difference, but to explore—descriptively—whether awareness, familiarity, or attitudes shift across the final stage of clinical training, when exposure to digital workflows and awareness of professional expectations are likely to intensify. Comparison by sex was similarly exploratory but was motivated by prior reports of sex-based differences in self-assessed competence with technical and digital tools—particularly artificial intelligence—in health-professional education, where male students often report higher self-efficacy despite comparable objective performance [14]. Examining whether such a pattern was present in this cohort was intended to inform whether curricular support for digital and AI-related skills may need to account for differences in self-perceived familiarity. The study was designed and reported in accordance with the Checklist for Reporting Results of Internet E-Surveys (CHERRIES) [15], and the completed checklist is provided as a supplementary file (Additional file 1).

Ethical approval was obtained from the Non-Invasive Clinical Research Ethics Committee of Dicle University Faculty of Dentistry before data collection (approval date: 25 June 2025; protocol no: 2025-55). The study was conducted in accordance with the principles of the Declaration of Helsinki. Participation was voluntary, and electronic informed consent was obtained from all participants before they completed the questionnaire. No personally identifying information was collected.

### Sample size and sampling method

This study was conducted at a single dental faculty to evaluate digital dentistry-related awareness, attitudes, and curriculum expectations within a clearly defined undergraduate educational setting. This design allowed the assessment of a relatively homogeneous group of clinical-year students who were exposed to the same curriculum and clinical training environment.

During the study period, the target population consisted of 220 clinical-year dental students, including 120 fourth-year and 100 fifth-year students. The sampling strategy was census-based rather than probability-based because all eligible students were invited to participate. Because a census of the eligible population was attempted, an a priori sample-size calculation was not applicable; instead, a post-hoc sensitivity analysis was conducted in G*Power 3.1.9.7 to determine the minimum effect size detectable with the achieved sample. With *N* = 187, α = 0.05, and power (1 − β) = 0.80, the study was sensitive to associations of Cramér’s V ≈ 0.25 for 2 × 5 contingency tables (df = 4) and V ≈ 0.21 for 2 × 2 tables — that is, small-to-medium effects. The nominally significant associations observed were of comparable magnitude (V ≈ 0.23–0.25), indicating that the sample was adequately powered to detect associations of the size encountered and that the predominantly null inferential findings are unlikely to reflect insufficient statistical power.

The questionnaire was created using Google Forms and distributed electronically through digital communication channels commonly used by dental students, such as student social media groups and WhatsApp groups. To prevent duplicate responses and ensure that each participant could only complete the questionnaire once, the survey parameters were set to restrict each email address to a single submission.

After excluding responses from students who did not provide electronic informed consent or failed the attention-check item, the final sample consisted of 187 students, corresponding to an overall response rate of 85.0%. Of these participants, 105 were fourth-year students and 82 were fifth-year students, representing response rates of 87.5% and 82.0%, respectively.

### Questionnaire development and validation

The questionnaire used in this study was developed by the research team following a comprehensive review of the contemporary literature on digital dentistry education, students’ awareness of digital technologies, and technology-related educational expectations in dentistry. Previously published survey-based studies evaluating dental students’ knowledge, awareness, perceptions, and attitudes toward digital dentistry and related digital technologies were examined during the questionnaire development process [16–19]. In addition, recently published studies investigating undergraduate dental students’ attitudes toward digital technologies and artificial intelligence in dental education were also considered during the conceptual structuring of the questionnaire [20, 21].

The questionnaire was designed to evaluate three principal domains: (i) self-reported awareness and familiarity with digital dentistry concepts and technologies, (ii) attitudes toward the integration of digital technologies into clinical practice and undergraduate dental education, and (iii) future professional perspectives regarding digital dentistry. This conceptual framework was established in accordance with commonly used knowledge–attitude–perception models in healthcare education research.

The questionnaire items were adapted from the reviewed literature and modified according to the aims and scope of the present study. The survey was initially prepared and administered in Turkish to ensure comprehensibility and to facilitate accurate responses from the participants. Following completion of the data collection process, the questionnaire content was translated into English for scientific reporting and publication purposes.

The final questionnaire consisted of 11 items organised into five thematic sections. The first section included an electronic informed consent item and an attention-check question designed to improve response validity in the online survey environment. The second section collected demographic information, including gender and year of study. The third section evaluated previous exposure to digital dentistry education and participants’ familiarity with digital dentistry technologies using a five-point familiarity scale. The fourth section assessed attitudes and perceptions regarding digital dentistry using five Likert-type statements rated on a five-point scale ranging from “strongly disagree” to “strongly agree.” The final section evaluated students’ perceptions regarding the adequacy of digital dentistry education at their faculty, future professional interest in digital dentistry, and expectations concerning the future role of digital technologies in dentistry.

Prior to distribution, the questionnaire items were reviewed by two clinicians with at least five years of experience in the relevant field to evaluate content relevance and clarity, and minor wording adjustments were made accordingly. To further document content validity, the final item set was additionally evaluated by an independent panel of seven experts (three from prosthodontics, two from oral and maxillofacial radiology, and two from orthodontics; all from different institutions and external to the study), who rated every item for relevance on a 4-point scale. Item-level content validity (I-CVI) met the ≥ 0.78 criterion recommended for seven raters for fourteen of the fifteen items. The only item below threshold was a reverse-worded barrier item (“learning digital systems is harder than traditional methods”; I-CVI = 0.57), which two experts independently noted measured perceived difficulty rather than attitude; this item was also flagged by the exploratory factor analysis and was excluded from the positive-attitude scale. The scale-level content validity index was S-CVI/Ave = 0.94 for the full item set and 0.97 for the final scale. The construct validity of the two multi-item scales (familiarity and attitude) was examined by exploratory factor analysis, and internal consistency was assessed using Cronbach’s α. Formal pilot testing of the instrument was not undertaken; this is acknowledged in the Limitations. The complete English version of the questionnaire is provided in Additional file 2: Table S1.

### Measurement tools

A five-point familiarity scale ranging from “Not familiar at all” to “Very familiar” was used to assess participants’ self-reported familiarity with digital dentistry technologies. Attitudes and perceptions regarding digital dentistry were evaluated using five-point Likert-type items (1 = Strongly disagree, 5 = Strongly agree) [22]. In addition, dichotomous (Yes/No), categorical, and multiple-choice items were included to evaluate participants’ demographic characteristics, previous educational exposure, educational expectations, and future professional perspectives regarding digital dentistry.

### Statistical analysis

Data were analysed using IBM SPSS Statistics version 27.0 (IBM Corp., Armonk, NY, USA). Descriptive statistics and frequency analyses were used to summarise participants’ demographic characteristics and questionnaire responses. Categorical variables were presented as frequencies and percentages. Internal consistency was assessed using Cronbach’s α; values were 0.828 for the familiarity scale and 0.92 for the positive-attitude scale (four items), and 0.793 for the original five-item attitude set.

Pearson’s chi-square test was used to analyse associations between categorical variables, including gender, year of study, and students’ responses to the questionnaire items. The magnitude of statistically significant or nominally significant associations was assessed using Cramér’s V as an effect-size estimate and interpreted as small (≈ 0.10), medium (≈ 0.30), or large (≈ 0.50). To account for multiple comparisons across questionnaire items, the Benjamini–Hochberg procedure was used to control the false discovery rate (FDR). Statistical significance was primarily interpreted according to FDR-adjusted p-values, with an adjusted p-value < 0.05 considered statistically significant. Nominal significance was defined as p-value < 0.05.

The construct validity and reliability analyses and the prior-exposure analyses were performed in jamovi (version 2.7; The jamovi project, Sydney, Australia), which provides integrated modules for exploratory factor analysis and non-parametric effect-size estimation. For each multi-item scale, factorability was confirmed using the Kaiser–Meyer–Olkin (KMO) measure and Bartlett’s test of sphericity, and exploratory factor analysis (minimum-residual extraction) was conducted, with the number of factors determined by the Kaiser criterion (eigenvalue > 1). A familiarity composite (mean of the five technology-specific items) and a positive-attitude composite (mean of the four attitude items that formed a single dimension) were then computed.

To examine whether prior exposure to digital dentistry education was associated with familiarity and attitudes, these composite scores were compared across exposure groups. Because the exposure subgroups were unequal in size and several item-level cross-tabulations contained cells with expected counts below five, non-parametric tests were used: the Mann–Whitney U test for the binary comparison (any prior exposure vs. none) and the Kruskal–Wallis test for the three-level comparison (yes / partial / no), with effect sizes reported as r (Z/√N) and ε². Item-level associations between prior exposure and individual items were additionally examined with the chi-square test and FDR correction, consistent with the main analyses. The three composite comparisons were pre-specified and interpreted at α = 0.05; for completeness, the familiarity associations also remained significant after FDR correction across these comparisons (adjusted *p* = 0.009 and 0.024).

## Results

### Participant characteristics

A total of 187 clinical-year dental students were included in the final analysis. Of these participants, 105 (56.1%) were fourth-year students and 82 (43.9%) were fifth-year students. Female students constituted 57.2% of the sample (*n* = 107), whereas male students accounted for 42.8% (*n* = 80). Most participants reported that they had not previously received education related to digital dentistry applications (73.8%), while 14.4% reported partial exposure and 11.8% reported having received such education (Table 1).

**Table 1:** Frequency distribution of responses to all survey items (*n* = 187)

Item	Main response pattern	*n*	%
Section 1. Demographic Information			
Gender	**Female**	**107**	**57.2**
	Male	80	42.8
Year of students	**4th year**	**105**	**56.1**
	5th year	82	43.9
Section 2. Prior Education and General Knowledge			
Prior training in digital dentistry	**No**	**138**	**73.8**
	Partially	27	14.4
	Yes	22	11.8
General familiarity with digital dentistry	**Limited knowledge**	**111**	**59.4**
	Moderate knowledge	63	33.7
	Good knowledge	9	4.8
	No knowledge (never heard)	4	2.1
Section 3. Familiarity with Specific Digital Technologies			
CAD/CAM systems	**Neutral**	**95**	**50.8**
	Familiar	60	32.1
	Not familiar	24	12.8
	Not familiar at all	6	3.2
	Very familiar	2	1.1
3D printers	**Neutral**	**94**	**50.3**
	Not familiar	46	24.6
	Familiar	35	18.7
	Not familiar at all	9	4.8
	Very familiar	3	1.6
Digital radiography systems	**Familiar**	**80**	**42.8**
	Neutral	61	32.6
	Not familiar	29	15.5
	Not familiar at all	9	4.8
	Very familiar	8	4.3
Intraoral scanners	**Neutral**	**82**	**43.9**
	Familiar	63	33.7
	Not familiar	34	18.2
	Not familiar at all	5	2.7
	Very familiar	3	1.6
Artificial intelligence applications	**Neutral**	**67**	**35.8**
	Not familiar	56	29.9
	Familiar	37	19.8
	Not familiar at all	21	11.2
	Very familiar	6	3.2
Section 4. Attitudes Toward Digital Dentistry			
Digital technologies increase efficiency in dental practice.	**Agree**	**94**	**50.3**
	Strongly agree	82	43.9
	Neutral	5	2.7
	Strongly disagree	4	2.1
	Disagree	2	1.1
Learning digital systems is harder than conventional methods.	**Disagree**	**75**	**40.1**
	Neutral	65	34.8
	Agree	24	12.8
	Strongly disagree	19	10.2
	Strongly agree	4	2.1
I have a high level of interest in digital applications.	**Agree**	**109**	**58.3**
	Strongly agree	47	25.1
	Neutral	24	12.8
	Strongly disagree	5	2.7
	Disagree	2	1.1
I would like to use digital technologies in clinical practice.	**Strongly agree**	**95**	**50.8**
	Agree	81	43.3
	Neutral	5	2.7
	Strongly disagree	4	2.1
	Disagree	2	1.1
I want digital technologies further integrated into my education.	**Strongly agree**	**106**	**56.7**
	Agree	71	38.0
	Strongly disagree	5	2.7
	Neutral	4	2.1
	Disagree	1	0.5
Section 5. Educational Environment and Future Intentions			
Digital dentistry is adequately taught at my faculty.	**No**	**139**	**74.3**
	Partially	40	21.4
	No opinion	5	2.7
	Yes	3	1.6
Future intentions to specialize in digital dentistry.	**Yes**,** I am considering**	**100**	**53.5**
	Yes, definitely	42	22.5
	Undecided	38	20.3
	No, I am not	7	3.7
Digital technologies will replace conventional dental methods.	**Yes**	**120**	**64.2**
	Partially	62	33.2
	No	5	2.7

Note. Bold values indicate the dominant response for each item. Likert scale: 1 = Strongly disagree / Not familiar at all; 5 = Strongly agree / Very familiar

### Questionnaire validation

Content validity was excellent: fourteen of the fifteen items met the I-CVI threshold, and the scale-level index was S-CVI/Ave = 0.94 (0.97 after exclusion of the reverse-worded barrier item).

Both multi-item scales were suitable for factor analysis (familiarity: KMO = 0.798, Bartlett’s χ²(10) = 385, *p* < 0.001; attitude: KMO = 0.811, Bartlett’s χ²(10) = 609, *p* < 0.001). For the familiarity scale, exploratory factor analysis yielded a single factor explaining 61.3% of the variance, with all five items loading strongly (0.54–0.83); Cronbach’s α was 0.828.

For the attitude items, exploratory factor analysis indicated that the reverse-worded item “Learning digital systems is harder than traditional methods” did not belong to the underlying construct: its loading was below 0.30 (uniqueness = 0.998) and its corrected item–total correlation was − 0.04, indicating that it captured perceived difficulty rather than positive attitude. This item was therefore treated as a standalone descriptive item and excluded from the attitude composite. The remaining four items formed a single, well-defined factor explaining 80.9% of the variance (loadings 0.77–0.94), with excellent internal consistency (Cronbach’s α = 0.92; α = 0.79 for all five items) (Table 2).

**Table 2:** Content validity and psychometric properties of the familiarity and attitude scales (*n* = 187)

Scale / item	I-CVI	Factor loading
*Familiarity scale* ^a^		
CAD/CAM systems	1.00	0.79
3D printers	1.00	0.79
Digital radiography systems	0.86	0.63
Intraoral scanners	1.00	0.83
AI applications	1.00	0.54
* Attitude scale* ^b^		
Improves efficiency	1.00	0.83
High interest	1.00	0.77
Willing to use clinically	1.00	0.94
Want more in education	1.00	0.93
Learning is harder (reverse-worded)	0.57	**< 0.30** ^c^

^a^KMO = 0.798; Bartlett’s χ²(10) = 385, *p* < 0.001; single factor, 61.3% of variance; Cronbach’s α = 0.828

^b^KMO = 0.811; Bartlett’s χ²(10) = 609, *p* < 0.001; single factor (four items), 80.9% of variance; Cronbach’s α = 0.92 (0.79 for five items)

ᶜThe bold factor loading (< 0.30) denotes an item that did not load on the attitude factor. This reverse-worded item (uniqueness = 0.998; corrected item–total r = −0.04) was excluded from the attitude composite

^d^S-CVI/Ave = 0.94 for the full 15-item set and 0.97 for the final scale. Content validity was assessed by an independent panel of seven experts; I-CVI ≥ 0.78 was considered acceptable

### Self-reported familiarity with digital dentistry technologies

Regarding general familiarity with the concept of digital dentistry, most participants reported limited or moderate knowledge. Among the specific technologies, digital radiography systems demonstrated the highest familiarity levels, whereas artificial intelligence applications showed the lowest familiarity levels among participants. For most technologies, neutral responses represented the dominant response category (Table 1).

### Attitudes and educational expectations toward digital dentistry

Participants generally demonstrated positive attitudes toward digital dentistry. Most students agreed that digital technologies improve efficiency in dental practice and expressed strong interest in digital applications. In addition, most participants reported willingness to use digital technologies in clinical practice and expressed a strong desire for greater integration of these technologies into undergraduate dental education. However, a substantial proportion of students considered digital dentistry education at their faculty to be insufficient (Table 1).

### Future perspectives toward digital dentistry

Most participants reported that they were considering incorporating digital dentistry into their future professional practice. In addition, most students believed that digital technologies would either completely or partially replace conventional dental methods in the future (Table 1).

### Gender-based comparisons

Gender-based comparisons showed a nominally significant association between gender and familiarity with artificial intelligence applications (χ² = 11.702, df = 4, unadjusted *p* = 0.020, Cramér’s V = 0.25). Male students appeared to report greater familiarity with artificial intelligence applications than female students. However, this association did not remain statistically significant after correction for multiple comparisons using the Benjamini–Hochberg procedure (FDR-adjusted *p* = 0.200).

No statistically significant gender differences were found for familiarity with CAD/CAM systems, 3D printers, digital radiography systems or intraoral scanners. Likewise, gender was not significantly associated with attitude-related items such as perceived efficiency of digital technologies, perceived difficulty of learning digital systems, interest in digital applications, willingness to use digital technologies in clinical practice, and desire for broader curricular integration of digital technologies in dental education (Table 3).

**Table 3:** Gender-based comparisons of familiarity and attitude-related questionnaire items (*N* = 187)

Questionnaire item	χ²	df	*p*-value	FDR-adjusted *p*-value	Cramér’s V	Interpretation
*Familiarity with Specific Technologies*						
CAD/CAM systems	3.800	4	0.434	0.531	0.14	Not significant
3D printers	7.914	4	0.095	0.391	0.21	Not significant
Digital radiographic systems	5.732	4	0.220	0.391	0.18	Not significant
Intraoral scanners	5.857	4	0.210	0.391	0.18	Not significant
Artificial intelligence applications	11.702	4	**0.020**	0.200	0.25	Nominally significant before FDR correction
*Attitudes Toward Digital Dentistry*						
Digital technologies increase efficiency in dentistry	3.502	4	0.478	0.531	0.14	Not significant
Digital systems are difficult to learn	0.506	4	0.973	0.973	0.05	Not significant
Interest in digital applications is high	5.256	4	0.262	0.391	0.17	Not significant
Willingness to use digital technologies in clinical practice	5.135	4	0.274	0.391	0.17	Not significant
Desire for greater inclusion of digital technologies in dental education	6.282	4	0.179	0.391	0.18	Not significant

Note. χ² = Pearson’s chi-square statistic; *df * degrees of freedom, *FDR * false discovery rate. FDR-adjusted *p*-values were calculated via the Benjamini–Hochberg procedure to correct for multiple comparisons. Adjusted *p* < 0.05 indicates statistical significance; unadjusted *p* < 0.05 indicates nominal significance. Cramér’s V estimates effect size (≈ 0.10 small, ≈ 0.30 medium, ≈ 0.50 large). Bold p-values indicate nominal significance (unadjusted p < 0.05) before FDR correction

### Year-of-study-based comparisons

Year-of-study-based comparisons initially revealed nominally significant associations between the academic year and two attitude-related items: willingness to use digital technologies in clinical practice (χ² = 10.103, df = 4, unadjusted *p* = 0.039, Cramér’s V = 0.23) and the desire for greater inclusion of digital technologies in dental education (χ² = 10.126, df = 4, unadjusted *p* = 0.038, Cramér’s V = 0.23). Fifth-year students demonstrated slightly more positive attitudes regarding these specific items compared to fourth-year students. However, these associations did not remain statistically significant after correcting for multiple comparisons via the Benjamini–Hochberg procedure (FDR-adjusted *p* = 0.195 for both items). Furthermore, no statistically significant differences were found between the fourth- and fifth-year cohorts regarding familiarity with CAD/CAM systems, 3D printers, digital radiographic systems, intraoral scanners, or artificial intelligence applications. Likewise, the academic year was not significantly associated with other perceptions, including the perceived efficiency of digital technologies, the perceived difficulty of learning digital systems, and general interest in digital applications (Table 4).

**Table 4:** Year-of-study-based comparisons of familiarity and attitude-related questionnaire items

Questionnaire item	χ²	df	*p*-value	FDR-adjusted *p*-value	Cramér’s V	Interpretation
*Familiarity with Specific Technologies*						
CAD/CAM systems	4.941	4	0.293	0.419	0.16	Not significant
3D printers	4.109	4	0.391	0.489	0.15	Not significant
Digital radiographic systems	5.570	4	0.234	0.419	0.17	Not significant
Intraoral scanners	5.309	4	0.257	0.419	0.17	Not significant
Artificial intelligence applications	1.902	4	0.754	0.754	0.10	Not significant
*Attitudes Toward Digital Dentistry*						
Digital technologies increase efficiency in dentistry	5.551	4	0.235	0.419	0.17	Not significant
Digital systems are difficult to learn	5.451	4	0.244	0.419	0.17	Not significant
Interest in digital applications is high	2.925	4	0.570	0.633	0.13	Not significant
Willingness to use digital technologies in clinical practice	10.103	4	**0.039**	0.195	0.23	Nominally significant before FDR correction
Desire for greater inclusion of digital technologies in dental education	10.126	4	**0.038**	0.195	0.23	Nominally significant before FDR correction

Note. χ² Pearson’s chi-square statistic, *df* degrees of freedom, *FDR* false discovery rate. FDR-adjusted *p*-values were calculated via the Benjamini–Hochberg procedure to correct for multiple comparisons. Adjusted *p* < 0.05 indicates statistical significance; unadjusted *p* < 0.05 indicates nominal significance. Cramér’s V estimates effect size (≈ 0.10 small, ≈ 0.30 medium, ≈ 0.50 large). Bold p-values indicate nominal significance (unadjusted p < 0.05) before FDR correction

### Prior exposure to digital dentistry education

Forty-nine students (26.2%) reported some prior exposure to digital dentistry education (22 [11.8%] “yes” and 27 [14.4%] “partial”), whereas 138 (73.8%) reported none.

Prior exposure was associated with higher self-reported familiarity. Students with any prior exposure scored higher on the familiarity composite than those without (median 3.20 [IQR 3.00–3.60] vs. 3.00 [2.60–3.40]; mean 3.22 ± 0.52 vs. 2.97 ± 0.71; Mann–Whitney U, *p* = 0.010, *r* = 0.19), with a consistent gradient across the three exposure levels (yes 3.35 ± 0.54, partial 3.12 ± 0.50, none 2.97 ± 0.71; Kruskal–Wallis H = 8.28, *p* = 0.016, ε² = 0.045). The same pattern was observed for general familiarity with the concept of digital dentistry (Kruskal–Wallis H = 11.69, *p* = 0.003, ε² = 0.063).

In contrast, attitudes did not differ by prior exposure. Positive-attitude composite scores were uniformly high across groups (any exposure 4.44 ± 0.48 vs. none 4.25 ± 0.78; Mann–Whitney U, *p* = 0.156, *r* = 0.10; three-level Kruskal–Wallis H = 2.46, *p* = 0.292, ε² = 0.013) (Table 5).

**Table 5:** Familiarity and attitude composite scores by prior exposure to digital dentistry education

Outcome (mean ± SD)	Yes (*n* = 22)	Partial (*n* = 27)	No (*n* = 138)	H	*p*	ε²
Familiarity composite	3.35 ± 0.54	3.12 ± 0.50	2.97 ± 0.71	8.28	0.016	0.045
General familiarity (1–4)	2.86 ± 0.71	2.41 ± 0.57	2.34 ± 0.59	11.69	0.003	0.063
Positive-attitude composite	4.50 ± 0.44	4.40 ± 0.52	4.25 ± 0.78	2.46	0.292	0.013

Kruskal–Wallis test across the three exposure groups; *df* = 2 for all comparisons. *ε²* = epsilon-squared effect size. Higher scores indicate greater familiarity / more positive attitude

At the item level, only the perception of faculty teaching adequacy was nominally associated with prior exposure (χ² = 9.02, *p* = 0.029); this did not remain significant after Benjamini–Hochberg correction (FDR-adjusted *p* = 0.30). No other item was significantly associated with prior exposure.

## Discussion

The aim of this study was to evaluate the awareness, attitudes and curriculum expectations of dental students in the clinical years towards digital dentistry at a single dental faculty in Türkiye. Most of the participants had limited formal exposure to digital dentistry as a defined curricular domain, but consistently showed positive attitudes to its clinical relevance and strong expectations for its wider integration into undergraduate education. These results point to a substantive mismatch between students’ positive orientation towards digital technologies and their self-reported familiarity with using them in a structured, competency-based manner – highly motivated students encountering an educationally underprepared environment. This pattern is consistent with international evidence that formal digital dentistry education lags behind both technological advancement and student expectations in multiple national contexts [1, 23–26].

### What the present study adds

While prior studies have investigated dental students’ awareness of or attitudes toward specific digital technologies—most commonly CAD/CAM systems [6] or intraoral scanners [27]—fewer have simultaneously assessed familiarity across multiple technology domains including AI applications [8], attitudes toward curricular integration [5], and future professional perspectives [1], within a clinical-year population. Most comparable studies have been conducted in Middle Eastern, North African, or Western European settings with varying degrees of digital infrastructure; evidence from Türkiye’s public dental faculty system specifically remains limited. The present study therefore offers context-specific data relevant to a country where the dental workforce is predominantly private-sector oriented and where the growth of international dental health tourism creates additional institutional pressure to produce digitally capable graduates [9, 10].

### Awareness and familiarity with digital dentistry technologies

The familiarity gradient observed in this study, with digital radiographic systems showing the highest familiarity, artificial intelligence applications the lowest, and CAD/CAM systems, 3D printing, and intraoral scanners clustering around neutral levels, suggests that students’ knowledge may largely reflect incidental clinical exposure rather than structured curricular training. Because digital radiography is routinely used in diagnosis and treatment planning, its relatively higher familiarity is expected. In contrast, technologies requiring dedicated workflows, laboratory infrastructure, or hands-on training appeared to remain at a more limited awareness level. This interpretation is supported by the finding that 73.8% of participants reported receiving no formal training in digital dentistry as a curricular domain.

The familiarity gradient observed here may also be shaped by institution-specific factors that influence how directly students engage with each technology. At the study site, digital content is integrated across existing courses rather than delivered as a standalone module, and the technologies differ in how far they form part of routine, student-performed clinical activity. Digital radiography is embedded in students’ day-to-day work, consistent with its comparatively high familiarity. Other modalities are encountered more indirectly: CBCT is typically used beyond the undergraduate scope of practice, while intraoral scanners, CAD/CAM systems, and 3D printing sit more within postgraduate or laboratory workflows, and artificial intelligence is met mainly through general-purpose tools rather than curriculum-embedded instruction. The familiarity gradient thus appears to track how far each technology is part of hands-on undergraduate practice at this institution rather than a uniform level of exposure—an interpretation to bear in mind when comparing these findings with institutions configured differently.

Previous studies point in the same direction. Zitzmann et al. described digital radiography as one of the few firmly established areas of digital dental education, whereas most other technologies remain unevenly integrated across institutions [23]. Afshar et al. found that even final year students did not have sufficient knowledge of digital dentistry, suggesting that progression through training alone does not generate competence [24]. The Turkish context appears no different: Gavgali and Kavaz found students and early-career dentists were mainly theoretically aware, with little practical opportunity and low self-rated competence [1]. In contrast, Schlenz et al. showed that hands-on CAD/CAM training improved students’ understanding and motivation for digital tools [28], suggesting that passive awareness rarely converts to practical competency without active educational intervention.

The low familiarity with artificial intelligence stands out. Yüzbaşıoğlu reported much the same gap—limited foundational AI knowledge among dental students, even though they wanted AI in the curriculum [29]. AI literacy therefore deserves a defined place in undergraduate teaching, framed as a set of specific competencies rather than general awareness. In dental education, these span several interconnected domains [30]: understanding how AI-based tools generate their outputs and where they fail; critically appraising and validating AI-generated findings against clinical and radiographic reference standards; recognising the ethical, medico-legal, and accountability implications of acting on algorithmic recommendations [8]; and appreciating data governance, patient privacy, and algorithmic bias. Embedding these within explicit curricular learning outcomes, rather than leaving them to incidental exposure, would position graduates as critical, accountable users of AI rather than passive recipients of automated output [30].

### Positive attitudes coexisting with perceived educational inadequacy

One of the most significant educational outcomes of this study was the coexistence of very positive attitudes towards digital dentistry and widespread dissatisfaction with current educational provision. Most participants agreed that digital technologies enhance clinical efficiency, showed strong interest in digital applications and reported willingness to utilize these tools in future clinical practice. However, 74.3% of respondents felt that the digital dentistry education provided by their faculty was insufficient and only 1.6% felt that it was adequate. This discrepancy indicates that student motivation is high but the educational scaffolding is still lacking.

This pattern can be explained through the Technology Acceptance Model, which points to perceived usefulness and perceived ease of use as important predictors of technology acceptance [31]. Although these constructs were not directly operationalised in the present study, the findings suggest that positive perceptions and intentions do not necessarily translate into competent use without structured training and educational support [32]. Students’ favourable attitudes may therefore reflect motivation and perceived relevance rather than demonstrated operational capability. Similar evidence has been reported by Afshar et al., who found a knowledge–attitude association among final-year Iranian dental students [24], and by Ciocan et al., who showed that students with sufficient digital design skills were more likely to judge institutional time allocation as adequate [26].

Together, these findings indicate that positive attitudes provide a valuable motivational foundation, but they require systematic curricular investment to develop into competency-based digital professionalism. Undergraduate education should therefore move beyond incidental exposure and intentionally build students’ capacity to engage critically with digital and AI-based tools and to address digital health equity [30].

### Technology-specific response patterns as curriculum planning evidence

Familiarity differed across technologies and broadly reflected the extent to which each tool was embedded in routine clinical practice. However, even digital radiography, the most clinically familiar technology, was rated as “familiar” by fewer than half of participants. This suggests that the main educational gap is not exposure alone, but the lack of structured progression from exposure to competence.

Curricular responses should therefore be differentiated by technology domain. For digital radiography, teaching could move beyond routine use toward image-quality assessment, diagnostic interpretation, radiation protection, and critical appraisal of automated outputs. For CAD/CAM systems, 3D printing, and intraoral scanners, where neutral responses predominated, students may benefit from structured hands-on training linked to clinical reasoning and evidence-based workflow selection. Artificial intelligence requires particular attention because it showed the lowest familiarity and should be integrated within a broader digital dentistry curriculum addressing output validation, clinical accountability, and responsible use.

A vertically integrated, competency-based approach may therefore be appropriate. Preclinical training could establish foundational digital literacy, while clinical-year education could provide supervised, case-based workflow training with explicit learning outcomes [30]. Without such structure, students’ motivation may remain shaped by informal exposure rather than evidence-based clinical judgement.

### Gender-based differences

Only AI familiarity showed a gender-based association, with male students reporting higher self-perceived familiarity than female students (unadjusted *p* = 0.020; Cramér’s V = 0.25). However, the effect was small, did not remain significant after FDR correction (adjusted *p* = 0.200), and was not observed for any other technology or attitude-related item. Therefore, this finding should be interpreted cautiously as an exploratory pattern rather than a categorical gender difference.

If this pattern reflects a real difference, it may relate more to informal engagement with AI than to differences in ability. Because AI was the least familiar technology and was not formally taught at the study institution, students’ exposure was likely shaped by self-directed or out-of-classroom learning. This interpretation is consistent with systematic reviews showing that healthcare students generally hold positive attitudes toward AI, while their baseline knowledge remains fragmented and often informally acquired [33, 34]. In such settings, male students may report higher AI self-efficacy [14], which could explain the small familiarity difference observed here. Interestingly, this was not the case for attitudes where male and female students showed similarities in positive attitudes toward digital dentistry as reported by Afshar et al. [24]. Formal teaching of AI may thus help to bridge informal gaps of familiarity and create a common basis, independent of gender.

### Year-of-study differences

Fifth-year students reported somewhat greater willingness to use digital technologies in clinical practice and a stronger desire for broader curricular integration than fourth-year students. These associations were nominally significant (unadjusted *p* = 0.039 and *p* = 0.038, respectively; Cramér’s V = 0.23) but did not remain significant after FDR correction (adjusted *p* = 0.195 for both), and should therefore be interpreted as exploratory. No year-based differences were observed in self-reported familiarity with any of the five technology domains, indicating that familiarity patterns were broadly consistent across cohorts.

Although tentative, this pattern is educationally relevant: students’ positive orientation toward digital dentistry may increase as graduation approaches, while their self-reported familiarity remains largely unchanged. This may suggest that clinical progression heightens awareness of the relevance of digital workflows without necessarily providing systematic training. Similar findings have been reported by Sheba et al., who found that students consistently perceived insufficient time allocation for digital training despite differences across year groups [19]. In Türkiye, Gavgali and Kavaz similarly reported more favourable attitudes at higher training levels despite limited hands-on exposure and insufficient curricular integration [1], while Bilici Geçer and Elburus found that perceptions of digital technologies varied by academic year [21].

These observations are more suggestive of a testable hypothesis than of a definitive conclusion. Introducing structured digital dentistry content earlier in clinical training, particularly in the fourth year, might help to build competence at a time when professional motivation is starting to increase. This possibility should be verified in longitudinal or multi-cohort studies.

### Prior exposure and the familiarity–attitude dissociation

Prior exposure to digital dentistry education was associated with greater self-reported familiarity, following a clear dose-response gradient, yet it was unrelated to attitudes, which remained uniformly positive regardless of previous exposure. This dissociation is informative: whereas familiarity appears to be experience-dependent and therefore responsive to curricular provision, the strong interest in digital dentistry was shared across the cohort, including students who had not yet received any related training. Motivation for digital dentistry thus does not appear to be contingent on prior exposure, reinforcing the view that the principal gap is one of provision rather than of student receptiveness. The association between prior exposure and higher familiarity scores additionally provides a form of known-groups (discriminant) validity for the familiarity scale, as the instrument distinguished groups expected a priori to differ.

### Future professional perspectives and curriculum development

Most participants intended to incorporate digital dentistry into their future clinical practice, and many expected digital technologies to partially or completely replace conventional methods. This suggests that clinical-year students increasingly view digital competence as part of their emerging professional identity. In the Turkish context, this perception is also shaped by the private-sector orientation of the dental workforce, the growing role of dental health tourism, and patient expectations for aesthetic, standardised, and time-efficient care. Consistent with this, Gavgali and Kavaz reported that many Turkish dental students and early-career dentists intended to use digital technologies in future practice, despite predominantly theoretical exposure and limited hands-on training [1].

The institutional response should therefore go beyond adding isolated digital content to the curriculum. Instead, competency-based frameworks are needed to define what clinical-year graduates should be able to do with digital tools, not only what they should know about them. Existing digital health curriculum frameworks similarly emphasise explicit competencies aligned with teaching and assessment, rather than assuming that exposure alone produces competence [30]. Although infrastructure and financial constraints in public dental faculties are real, incremental strategies such as shared digital facilities, faculty development, and partnerships with technology providers may offer feasible pathways toward expanded digital dentistry education.

### Strengths and limitations

There are particular methodological strengths of this study. The focus on fourth- and fifth-year students means that findings are representative of the transitional period in which undergraduate training begins to meet the demands of imminent professional practice. The simultaneous evaluation of five technology domains, including artificial intelligence, provided a broader familiarity profile than surveys limited to a single technology. The census-based sampling strategy and high response rate (85.0%) also support the internal validity of the findings within the target population. Finally, the questionnaire was supported by a multi-step validation process—expert-panel content validity assessment (S-CVI/Ave = 0.94), exploratory factor analysis, internal-consistency reliability, and known-groups validity—providing a stronger psychometric basis than is typical for survey-based studies in this field.

Several limitations should nonetheless be acknowledged. First, the single-institution design inherently limits the generalisability of the findings. The results should be understood primarily as institution-specific observations: patterns such as the high overall enthusiasm or the particular ordering of technology familiarity may reflect the specific curriculum, clinical infrastructure, and resources at this institution rather than dental education more broadly. The broader educational implications drawn here are therefore best regarded as hypotheses requiring confirmation rather than directly generalisable conclusions, and multi-institutional replication will be necessary to establish how widely these patterns apply. Second, the study evaluated self-reported familiarity and attitudes rather than objective knowledge or practical skill. Self-perceived familiarity does not necessarily correspond to measurable digital literacy or clinical proficiency; a student may feel familiar with a technology without being able to apply it competently, or conversely may underrate genuinely developed skills. The findings should therefore be read as reflecting students’ perceptions and self-appraisals rather than their actual competence, and future research should incorporate objective knowledge assessments and, where feasible, performance-based clinical evaluation to bridge this gap. Third, the cross-sectional design precludes causal inferences regarding how students’ attitudes develop over time. Fourth, although the instrument underwent a multi-step validation process, it was not formally pilot-tested, and confirmatory factor analysis in independent samples across diverse educational settings has not yet been performed. Fifth, recruitment through student social media and messaging groups may have introduced some self-selection toward more digitally engaged students. In addition, the survey did not address several emerging technologies, such as augmented or virtual reality, dynamic implant navigation, digital smile design, and robotic-assisted procedures, and the analysis remained descriptive and bivariate. Future studies should include multivariable modelling and longitudinal designs to determine whether students’ positive intentions translate into sustained clinical adoption.

## Conclusion

This study demonstrates that clinical-year students at a Turkish public dental faculty hold consistently positive professional attitudes toward digital dentistry and strong expectations for its curricular integration, while their formal preparation and self-reported familiarity remain limited across most technology domains, with artificial intelligence applications the least understood. The absence of significant gender- or year-based differences after correction for multiple comparisons suggests that this gap is broadly shared across the cohort rather than confined to a particular subgroup. The central implication is not that students are under-motivated to engage with digital dentistry, but that their motivation currently exceeds the educational infrastructure available to channel it toward functional competence. With institutional support, the enthusiasm displayed in this study can be translated into the digital professionalism that modern dental practice requires more and more.

## Supplementary Information

Additional file 1: CHERRIES checklist. Completed Checklist for Reporting Results of Internet E-Surveys for the present study.

Additional file 2: Table S1. English version of the questionnaire used in the study.

## Data Availability

The datasets generated and analysed during the current study are not publicly available in order to protect participant confidentiality but are available from the corresponding author on reasonable request.

## References

[1] Gavgali YB, Kavaz T. Future of dental education: digitalization, career outlook, and perspectives of dental students and early-career dentists. BMC Med Educ. 2026;26:40. 10.1186/s12909-025-08467-y. PMC12784569 41437250

[2] Alauddin MS, Baharuddin AS, Mohd Ghazali MI. The modern and digital transformation of oral health care: A mini review. Healthcare (Basel). 2021;9(2):118. 10.3390/healthcare9020118. PMC7912705 33503807

[3] Ohyama H, Duong M-L, Yancoskie AE, Smiley AB, Syed AZ, Reddy MS, et al. Challenges and opportunities in implementing digital technology in dental curriculum: a review and perspective. Cureus. 2025;17(4):e83272. 10.7759/cureus.83272. PMC12125001 40453304

[4] Oliveira ML. Digital Dental Radiology and Diagnostics—From 2D to 3D. Aust Dent J. 2025;70(S1):S50–S66. 10.1111/adj.70024. PMC12747602 41277722

[5] Liu C-M, Hsu M-H, Ng MY, Yu C-H. Digital integration in dental education: A novel self-directed learning model using intraoral scanners for tooth preparation training. J Dent Sci. 2025;20:639–45. 10.1016/j.jds.2024.10.030 PMC11762640 39873029

[6] Kiršić SP, Rajčić P, Vujević M, Udiljak Ž. Knowledge, Attitudes and Practical Experience With CAD / CAM Technology Among Dental Students in Croatia: A Cross-Sectional Study. Eur J Dent Educ. 2026. 10.1111/eje.70126.41777077

[7] Sahrir CD, Wang C-W, Shen Y-K, Lin W-C. Three-Dimensional Printing in Dentistry: Evolution, Technologies, and Clinical Application. Polymers. 2026;18:785. 10.3390/polym18070785 PMC13074545 41977533

[8] Németh T, Irandoust S, Dávidovics A. AI in medical and dentistry education: perspectives from international students, educators and physicians. BMC Med Educ. 2026;26:534. 10.1186/s12909-026-08886-5. PMC13040955 41742210

[9] Turkish Dental Association. Distribution of Dentists by Employment Type in Institutions, Provinces, and Districts in 2024. Turkish Dental Association. 2025. https://www.tdb.org.tr/tdb/v2/yayinlar/Dishekimi_Dagilim_Kitapciklari/2024YiliDishDagKitapcigi.pdf. Accessed 11 May 2026.

[10] Republic of Türkiye Ministry of Health, Department of Health Tourism. Authorized Health Facilities. 2026. https://shgmturizmdb.saglik.gov.tr/TR-76664/yetkili-saglik-tesisleri.html. Accessed 11 May 2026.

[11] Gawali N, Shah PP, Gowdar IM, Bhavsar KA, Giri D, Laddha R. The evolution of digital dentistry: a comprehensive review. J Pharm Bioallied Sci. 2024;16(Suppl 3):S1920–2. 10.4103/jpbs.jpbs_11_24 PMC11426768 39346228

[12] Chawla R. Systematic Literature Review on Dental Tourism: Travel with a Smile. J Int Oral Health. 2025;17:461–7.

[13] Keleş ZH, Ertuğrul MO, Tunçer J. Investigation of factors influencing patients’ preferences for traveling to Türkiye for dental tourism: The case of a private hospital. Anatol Clin J Med Sci. 2026;31:117–27.

[14] Laupichler MC, Aster A, Meyerheim M, Raupach T, Mergen M. Medical students’ AI literacy and attitudes towards AI: a cross-sectional two-center study using pre-validated assessment instruments. BMC Med Educ. 2024;24:401. 10.1186/s12909-024-05400-7. PMC11007897 38600457

[15] Eysenbach G. Improving the quality of Web surveys: the Checklist for Reporting Results of Internet E-Surveys (CHERRIES). J Med Internet Res. 2004;6:e34. 10.2196/jmir.6.3.e34 PMC1550605 15471760

[16] Sharab L, Adel M, Abualsoud R, Hall B, Albaree S, De Leeuw R, et al. Perception, awareness, and attitude toward digital dentistry among pre-dental students: an observational survey. Bull Natl Res Cent. 2022;46:246. 10.1186/s42269-022-00937-3.

[17] Umer MF, Faheemuddin M, Alshehri YA, Alqosaibi AA, Alojail AA, Althani AA, et al. Assessment of digital dentistry knowledge and practices among dental students at King Faisal university, Saudi Arabia. Med Sci Monit. 2024;30:e944692. 10.12659/MSM.944692 PMC11302256 39004882

[18] Madfa AA, Al-Ibrahim IK, Alshammary FL, Alharbi SM, Alharbi BB. An audit of Saudi dental students’ opinions and attitudes toward digital dentistry. Open Dentistry J. 2023;17:e18742106276082. 10.2174/0118742106276082231123053341.

[19] Sheba M, Comnick C, Elkerdani T, Ashida S, Zeng E, Marchini L. Students’ perceptions and attitudes about digital dental technology is associated with their intention to use it. J Dent Educ. 2021;85:1427–34. 10.1002/jdd.12610. 33864265

[20] Fitzek S, Choi K-EA. Shaping future practices: German-speaking medical and dental students’ perceptions of artificial intelligence in healthcare. BMC Med Educ. 2024;24:844. 10.1186/s12909-024-05826-z. PMC11304766 39107732

[21] Bilici Geçer R, Elburus B. Awareness and attitudes toward digital technologies in orthodontics among dental students in Turkey: a cross-sectional study. BMC Med Educ. 2025;25:1380. 10.1186/s12909-025-08017-6. PMC12512987 41068807

[22] Likert R. A technique for the measurement of attitudes. Arch Psychol. 1932;22(140):5–55.

[23] Zitzmann NU, Matthisson L, Ohla H, Joda T. Digital undergraduate education in dentistry: a systematic review. Int J Environ Res Public Health. 2020;17:3269. 10.3390/ijerph17093269 PMC7246576 32392877

[24] Afshar MK, Afshar MK, Elmamooz N, Ahmadi AG. Assessment of Knowledge and Attitudes Toward Digital Dentistry among Final-Year Dental Students in Southeast Iran: A Cross-Sectional Study. Pesquisa Brasileira em Odontopediatria e Clínica Integrada. 2026;26:e250016.

[25] Hall MA, Karawia I, Mahmoud AZ, Mohamed OS. Knowledge, awareness, and perception of digital dentistry among Egyptian dentists: a cross-sectional study. BMC Oral Health. 2023;23:963. 10.1186/s12903-023-03698-1. PMC10694936 38049790

[26] Ciocan LT, Pantea M, Vasilescu VG, Țâncu AMC, Sfeatcu R, Didilescu AC, et al. Evaluation of undergraduate dental students’ opinions on the use of digital versus conventional design in prosthodontics. Dentistry J. 2025;13:242. 10.3390/dj13060242 PMC12192495 40559145

[27] Lam WY-H, Mak KC-K, Maghami E, Molinero-Mourelle P. Dental students’ preference and perception on intraoral scanning and impression making. BMC Med Educ. 2021;21:501. 10.1186/s12909-021-02894-3. PMC8456611 34551730

[28] Schlenz MA, Michel K, Wegner K, Schmidt A, Rehmann P, Wöstmann B. Undergraduate dental students’ perspective on the implementation of digital dentistry in the preclinical curriculum: a questionnaire survey. BMC Oral Health. 2020;20:78. 10.1186/s12903-020-01071-0. PMC7079522 32188456

[29] Yüzbaşıoğlu E. Attitudes and perceptions of dental students towards artificial intelligence. J Dent Educ. 2021;85:60–8. 10.1002/jdd.12385. 32851649

[30] Mun M, Byrne S, Shaw L, Lyons K. Digital dentists: a curriculum for the 21st century. JMIR Med Educ. 2025;11:e54153. 10.2196/54153 PMC11735848 39789757

[31] Davis FD. Perceived usefulness, perceived ease of use, and user acceptance of information technology. MIS Q. 1989;13:319–40. 10.2307/249008.

[32] Bahanan L, Alsharif M. Factors affecting the acceptance of teledentistry determined using the technology acceptance model: A cross-sectional study. Digit Health. 2023;9:20552076231158034 10.1177/20552076231158034 PMC9941601 36825078

[33] Amiri H, Peiravi S, Rezazadeh Shojaee SS, Rouhparvarzamin M, Nateghi MN, Etemadi MH, et al. Medical, dental, and nursing students’ attitudes and knowledge towards artificial intelligence: a systematic review and meta-analysis. BMC Med Educ. 2024;24:412. 10.1186/s12909-024-05406-1. PMC11017500 38622577

[34] Mousavi Baigi SF, Sarbaz M, Ghaddaripouri K, Ghaddaripouri M, Mousavi AS, Kimiafar K. Attitudes, knowledge, and skills towards artificial intelligence among healthcare students: A systematic review. Health Sci Rep. 2023;6:e1138. 10.1002/hsr2.1138. PMC10009305 36923372

