# Supplementary material for: Digital dentistry among clinical-year dental students: awareness, attitudes, and curriculum expectations
Source: BMC Med Educ. 2026 Jul 15;26:1632. doi: 10.1186/s12909-026-09901-5 (PMC13660230; doi:10.1186/s12909-026-09901-5)
Supplement: Supplementary file 1 [file 12909_2026_9901_MOESM1_ESM.docx]

**Additional file 1. Completed CHERRIES Checklist**

*Checklist for Reporting Results of Internet E-Surveys (CHERRIES) for the present study.*

| **Checklist item** | **How addressed in this study** |
| --- | --- |
| **Design** | |
| Describe survey design | Descriptive cross-sectional online survey of fourth- and fifth-year (clinical-year) dental students at a single dental faculty (Dicle University Faculty of Dentistry, Türkiye). A census-based approach was used, inviting the entire eligible target cohort rather than a sample. This was a closed survey (restricted to the defined student cohort), not an open public survey. |
| **IRB approval and informed consent process** | |
| IRB approval | Approved by the Non-Invasive Clinical Research Ethics Committee of Dicle University Faculty of Dentistry (protocol no. 2025-55; approval date 25 June 2025). Conducted in accordance with the Declaration of Helsinki. |
| Informed consent | Participation was voluntary. The first questionnaire item required participants to confirm voluntary participation before proceeding; responses from participants who did not consent were excluded. Responses were anonymous and used exclusively for scientific research purposes. |
| Data protection | No personally identifying information was collected; all responses were anonymous. Data were stored within the password-protected survey platform accessible only to the research team. |
| **Development and pre-testing** | |
| Development and testing | The questionnaire was developed by the research team based on a review of the contemporary literature on digital dentistry education. Before distribution, the study team reviewed the instrument for linguistic clarity, content appropriateness, and comprehensibility for clinical-year dental students. No separate pilot study was conducted. |
| **Recruitment process and description of sample** | |
| Open survey vs closed survey | Closed survey: distributed only to the known, defined cohort of clinical-year dental students; not open to the general public. |
| Contact mode | Initial contact with potential participants was made online. |
| Advertising the survey | The survey link was distributed through student social-media and messaging groups used by the target cohort. No paid advertising or banner ads were used. |
| **Survey administration** | |
| Web/E-mail | Web-based survey administered via Google Forms; responses were captured automatically into the platform database. |
| Context | Hosted on Google Forms and shared within closed student groups of the target cohort. It was not posted on any public-content website, minimising content-driven self-selection. |
| Mandatory/voluntary | Voluntary. |
| Incentives | No incentives (monetary or non-monetary) were offered. |
| Time/Date | Data were collected between September and October 2025. |
| Randomization of items or questionnaires | Items were not randomized; all respondents received items in a fixed order. |
| Adaptive questioning | Adaptive questioning was not used; all respondents were presented with the same items. |
| Number of items | 11 items in total. |
| Number of screens (pages) | The questionnaire was distributed over 5 sections (pages). |
| Completeness check | All items were set as mandatory ('required') in Google Forms; the questionnaire could not be submitted with unanswered items, ensuring complete responses. In addition, an attention-check item ('please select "Usually"') was included; responses failing the attention check were excluded prior to analysis. |
| Review step | Respondents were able to review and change their answers before final submission (Google Forms 'Back' navigation was enabled). |
| **Response rates** | |
| Unique site visitor | Not applicable. This was a closed survey distributed to a defined cohort rather than an open website with site visitors. |
| View rate | Not applicable (closed survey; no public survey landing page with site-visitor traffic). |
| Participation rate | Of the defined eligible cohort, 187 students provided analysable responses, corresponding to a participation/completion rate of 85.0% among the invited target population. |
| Completion rate | Because all items were mandatory, all submitted questionnaires were complete; the analysed sample comprised 187 valid responses after exclusion of non-consenting and attention-check-failing participants. |
| **Preventing multiple entries from same individual** | |
| Cookies used | Cookies were not used to identify respondents. |
| IP check | IP addresses were not used to identify duplicate entries. |
| Log file analysis / duplicate prevention | To prevent duplicate responses and ensure that each participant could complete the questionnaire only once, the survey parameters were set to restrict each email address to a single submission. |
| **Analysis** | |
| Handling of incomplete questionnaires | All items were mandatory, so incomplete submissions did not occur. Responses from participants who did not consent or who failed the attention-check item were excluded prior to analysis (final analysed n = 187). |
| Questionnaires submitted with an atypical timestamp | Timestamp/outlier analysis was not performed. |
| Statistical correction | Multiple-comparison correction was applied using the Benjamini–Hochberg false-discovery-rate procedure; Cramér's V was reported as an effect-size estimate. |
