# Supplementary material for: Digital dentistry among clinical-year dental students: awareness, attitudes, and curriculum expectations
Source: BMC Med Educ. 2026 Jul 15;26:1632. doi: 10.1186/s12909-026-09901-5 (PMC13660230; doi:10.1186/s12909-026-09901-5)
Supplement: Supplementary file 2 [file 12909_2026_9901_MOESM2_ESM.docx]

***Table 1. Survey Instrument — Items, Response Types, and Response Options***

| **No.** | **Survey Item** | **Item Type** | **Response Options / Sub-Items** |
| --- | --- | --- | --- |
| **SECTION 1: Informed Consent and Attention Check** | | | |
| **1** | **Do you confirm that you are participating in this survey voluntarily?** | Single choice | Yes / No |
| **2** | **This item is designed to assess the respondent's level of attention. Please select the option "Usually."** | Single choice (Attention check) | Always / Sometimes / Usually / Rarely / Never |
| **SECTION 2: Demographic Information** | | | |
| **3** | **What is your gender?** | Single choice | Female / Male |
| **4** | **What year are you currently in?** | Single choice | 4th Year / 5th Year |
| **SECTION 3: Prior Education and Knowledge Level** | | | |
| **5** | **Have you previously received any training that includes digital dentistry applications?** | Single choice | Yes / No / Partially |
| **6** | **How familiar are you with the concept of digital dentistry?** | Single choice | I have never heard of it / I have some knowledge / I have moderate knowledge / I am knowledgeable / I am very knowledgeable |
| **7** | **How familiar are you with the following digital technologies?**  CAD/CAM systems  3D printers  Digital radiography systems  Intraoral scanners  Artificial intelligence applications | 5-point Likert scale | Not familiar at all / Not familiar / Neutral / Familiar / Very familiar |
| **SECTION 4: Attitudes and Perceptions** | | | |
| **8** | **To what extent do you agree with the following statements?**  1. Digital technologies enhance efficiency in dental practice.  2. Learning digital systems is more difficult than learning conventional methods.  3. I have a high level of interest in digital applications.  4. I would like to use digital technologies in clinical practice.  5. I believe digital technologies should be more extensively integrated into my education. | 5-point Likert scale | Strongly Disagree / Disagree / Neutral / Agree / Strongly Agree |
| **SECTION 5: Educational Environment and Future Intentions** | | | |
| **9** | **In your opinion, are digital dentistry applications adequately taught at your faculty?** | Single choice | Yes / No / Partially / I have no opinion |
| **10** | **Are you considering specializing in the field of digital dentistry in the future?** | Single choice | Yes, definitely / Yes, I am considering it / Undecided / No, I am not considering it |
| **11** | **Do you think digital technologies will replace conventional methods in dentistry in the future?** | Single choice | Yes / Partially / No / I have no opinion |

*Note. Target population: 4th- and 5th-year dental students. All responses are anonymous and will be used exclusively for scientific research purposes.*
